# No association between polymorphisms in the promoter region of dopamine receptor D2 gene and schizophrenia in the northern Chinese Han population: A case–control study

**DOI:** 10.1002/brb3.1193

**Published:** 2019-01-18

**Authors:** Xi‐cen Zhang, Mei Ding, Atif Adnan, Yi Liu, Yong‐ping Liu, Jia‐xin Xing, Jin‐feng Xuan, Xi Xia, Jun Yao, Bao‐jie Wang

**Affiliations:** ^1^ School of Forensic Medicine China Medical University Shenyang China

**Keywords:** association, dopamine, promoter region, schizophrenia

## Abstract

**Background:**

Epidemiological studies found that genetic factors are among the causes of schizophrenia, exclusively the genes involved in the dopamine system. Prior to this, the role of dopamine receptor D2 (*DRD2*) gene promoter polymorphisms and schizophrenia has been studied extensively, but there are still some uncertainties about these associations. The present study is focusing on the association between the *DRD2* gene promoter region polymorphisms and schizophrenia in the northern Chinese Han population.

**Methods:**

We sequenced 2,111‐bp fragment of *DRD2* gene promoter region in 306 schizophrenic patients and 324 healthy controls to find association between *DRD2* and schizophrenia. SPSS version 18 0.0 was used to calculate odds ratios (OR), 95% confidence intervals (CIs).The Hardy–Weinberg equilibrium test and the confirmation of haplotypes were calculated using Haploview version 4.1. The association of schizophrenic risk of *DRD2* genotypes, alleles, and haplotypes between case and control groups was calculated using the chi‐squared test. PS program was used to calculate the Power analysis.

**Results:**

The genotype frequencies of rs7116768 (*p* = 0.025) and rs1799732 (*p* = 0.042) were associated meagerly. After Bonferroni correction, there was no association found between *DRD2* gene promoter region with schizophrenia risk in the northern Chinese Han population.

**Conclusions:**

In this study, we did not find any significant difference between schizophrenia and the polymorphisms of *DRD2* gene promoter region. A more forceful conclusion remains to be verified by further confirmatory experiments.

## 1 INTRODUCTION

Schizophrenia is a multifactorial common psychiatric disorder (Casey, Rodriguez, Northcott, Vickar, & Shihabuddin, 2011), in which both environmental and genetic factors play an important role. In Schizophrenia degree of inheritance, involvement may be more than 80% (Sullivan, Kendler, & Neale, 2003). Different hypotheses were created to explain the pathogenesis of schizophrenia and propose that these interact to funnel through one final common pathway of presynaptic striatal hyperdopaminergia. (Howes & Kapur, 2009). Dopamine receptors are proteins that bind to dopamine specifically. Mutations in the genes may lead to the changes of their expression or their ability to bind to dopamine which initiates the process of schizophrenia. The dopamine receptor D2 (*DRD2*) gene is located in 11q22~23 extends over 270 kb and contains eight exons and seven introns. Long use of classic antipsychotic medications, which may preferentially antagonize dopamine receptors. Previous studies which includes the autopsy results of patients with schizophrenia (Camps, Cortes, Gueye, Probst, & Palacios, 1989; Hess, Bracha, Kleinman, & Creese, 1987), findings from PET studies (Breier et al., 1997; Laruelle et al., 1996, 1997), and pharmacological evidence (Seeman, 2002; Worrel, Marken, Beckman, & Ruehter, 2000) suggested a close relationship for *DRD2* gene and schizophrenia.

Up till now, most studies related to the association between the promoter region in *DRD2* gene and schizophrenia mainly focused on rs1799732. Arinami, Gao, Hamaguchi, and Toru (1997) suggested that the del C allele in schizophrenic patients can reduce the level of the expression of *DRD2* gene encoding area by 20%~40%. Subsequently, the following studies did not find a consistent conclusion in different geographical areas, such as England (Breen et al., 1999), Spain (Doehring, Kirchhof, & Lotsch, 2009), and in Japan (Hori, Ohmori, Shinkai, Kojima, & Nakamura, 2001). Ikeda et al. (2008) reported that rs1799978 is associated with the treatment and drug reaction against schizophrenia. The C allele of rs12364283 is associated with enhanced transcription and increased density of D2 receptors (Bertolino et al., 2009; Zhang et al., 2007). However, to fully explore the association of *DRD2* gene and schizophrenia, more and more studies are required for better understanding.

In the current study, we try to explore whether the polymorphisms in the promoter region of *DRD2* gene was involved in schizophrenia among the northern Chinese Han population.

## 2 MATERIALS AND METHODS

### 2.1 Study subjects

In this study, 306 schizophrenic patients and 324 healthy subjects were recruited. The schizophrenia group (*n* = 306) which came from the Third People's Hospital of Liaoning Province comprised of 154 males and 152 females, and the control group (*n* = 324) included 157 males and 167 females. The control samples were provided by China Medical University, and the individuals with any mental illness and other serious diseases were excluded. The mean age of patients was 44.6 ± 7.3 (mean ± standard deviation) years, and the mean age of healthy subjects was 45.3 ± 15.9 years. The mean onset age of case group was 25.68 ± 5.12. The disease duration was 5–23 years. The patients included were all paranoid schizophrenia. All patients were assessed for age at first hospitalization, first degree relatives with a history of mental illness, alcohol or drug abuse, antipsychotic reactions to schizophrenia, suicide attempts, and anticholinergic medication. Only patients who fully meet the diagnostic and statistical manual of mental disorders (fourth edition) were included in this study, which were diagnosed by expert professionals. Each of the subjects signed a written informed consent form before participating in this study. Sample collection and analysis have been approved by the Ethics Committee of China Medical University.

### 2.2 DNA extraction

Peripheral blood samples collected from each subject were stored in the EDTA tube. Genomic DNA was extracted applying phenol–chloroform method (Kramvis, Bukofzer, & Kew, 1996).

### 2.3 Segment selection and primer designing

In order to detect polymorphisms which have more possibility to impact the expression of *DRD2* gene, we chose a 2,111‐bp fragment upstream of the 5′ untranslated region (5'UTR) in *DRD2* gene. The fragment is closest to the coding region, and the polymorphisms within the fragment are more likely to affect gene expression. Sequencing primers were designed using the Primer Premier 5 Design Program (www.premierbiosoft.com). The sense and antisense primers used by PCRs were 5′‐CAACCATATCTGTAATGGCTGATCC‐3′ and 5′‐CTTCTAAGTGGCGAGGAGGCTAC‐3′, respectively.

### 2.4 Polymerase chain reaction amplification

Polymerase chain reactions were performed on TaKaRa PCR Thermal Cycler Dice™ (TP650) system (Japan) in a 20 µl reaction volume contains 4 µl 5× PrimeSTAR buffer, 2 µl dNTPs, 1.4 µl DMSO, 3 µl 15× each primer, 0.3 µl of PrimeStar HS polymerase 0.75 U (TaKaRa, Japan), and 3 µl of genomic DNA. The PCR condition was predenaturation at 98°C for 5 min, denaturation temperature of 98°C for 10 s, annealing temperature of 60.5°C for 5 s, extension temperature of 72°C for 2 min, and the number of cycles was 30.

### 2.5 Sequencing and alignment

The PCR products of around 2000 bp size were sent to the Taihe Biotechnology Co. (Beijing, China) Because of the technical limitations, it was not possible to sequence 2000 bp so we sequenced it in several segments. Primer details are in Table 1.

**Table 1:** Sequencing primers of *DRD2* gene

Primer	Sequence 5′−3′
A	CAACCATATCTGTAATGGCTGATCC
B	GGCGGTCGAGGGTTGCGTTCC
C	AGACCTGAAGTCAGAAAACG
D	GGAGTGGCCGCACAAACTTCTGGTC

After successful sequencing, we aligned the sequenced sequences with the reference sequences which had been reported in the National Center for Biotechnology Information database (https://www.ncbi.nlm.nih.gov/gene/) to identify polymorphisms.

### 2.6 Statistical analysis

Odds ratios (OR) and 95% confidence intervals (CIs) were calculated by SPSS 18.0 software (IBM, Armonk, NY, USA). The Hardy–Weinberg equilibrium test and the haplotype verification were evaluated using Haploview 4.1 software (Broad Institute, Cambridge, MA, USA). The chi‐square test was used to assess the associations between alleles, genotypes, haplotypes, and risk of schizophrenia, respectively. Bonferroni correction was used in multiple independent tests (*p* < 0.0125 was statistically significant). PS program (Dupont & Plummer, 1998) was used to calculate the Power analysis.

## 3 RESULTS

### 3.1 Genotype analysis

We detected six SNPs (rs7116768, rs1047479195, rs1799732, rs1799978, rs12364283, and rs80202441) through the analysis of sequencing results. Allele frequencies and genotype frequencies of the detected SNP loci are listed in Table 2.

**Table 2:** Genotype and allele frequencies of *DRD2* SNPs in control subjects and schizophrenia patients

SNP	Case		Control		*p*‐Value	OR	95% CI	Power
	*N*	%	*N*	%				
Rs7116768					0.025			
G/G	257	83.99	275	84.88				
G/C	33	10.78	44	13.58				
C/C	16	5.23	5	1.54				
G/G + G/C	290	94.77	319	98.46	0.013	0.284	0.103–0.785	0.729
C allele	65	10.5	54	8.3	0.178	1.307	0.895–1.910	0.283
Rs1047479195					0.403			
C/C	284	92.81	306	94.44				
C/A	20	6.54	14	4.32				
A/A	2	0.65	4	1.23				
A/A + C/A	22	7.19	18	5.55	0.418	1.317	0.692–2.506	0.135
A allele	24	3.9	22	3.4	0.654	1.161	0.644–2.094	0.079
Rs1799732					0.042			
Ins/Ins	257	83.99	275	84.88				
Ins/Del	34	11.11	44	13.58				
Del/Del	15	4.90	5	1.54				
Ins/Ins + Ins/Del	291	95.1	319	98.46	0.021	0.304	0.109–0.847	0.669
Del allele	64	10.5	54	8.3	0.209	1.285	0.878–1.879	0.253
Rs1799978					0.120			
A/A	209	68.30	208	64.20				
G/A	83	27.12	87	26.85				
G/G	14	4.58	28	8.64				
A/A + G/A	292	95.42	296	91.36	0.054	1.973	1.018–3.823	0.534
G allele	111	18.14	143	22.92	0.092	0.782	0.593–1.032	0.423

Note

The SNPs with minor allele frequency <0.01 were excluded. The *p*‐value was calculated by 2 × 3 and 2 × 2 chi‐square test, in which the codominant model, the recessive model, and the allele model were corrected by Bonferroni's correction and the *p < 0.05/4*was statistically significant. The statistical power is considered to be enough to detect any significant difference when power >0.8. The false discovery rate <0.05.

Among six SNPs, four SNPs (rs12364283, rs1799732, rs7116768, and rs8020241) were in HWE in control groups (*p* > 0.05). The frequencies of rs12364283 and rs80202441 were too low to carry out a statistical analysis (the minor allele frequencies were 0.003 and 0.006, respectively). Rs1799978 and rs1047479195 were not associated with schizophrenia (*p*‐value was 0.120 and 0.403, respectively). However, the genotype frequencies of rs7116768 and rs1799732 had a relevance to the occurrence of schizophrenia (*p*‐value was 0.025 and 0.042, respectively). However, when a Bonferroni correction was applied to mitigate against the so‐called “multiple comparison problem” (where for a significant *p*‐value of 0.5, 5% of tests are likely to be significant by chance), no significances were found. There was no significant difference in the allele frequencies of each site between the case group and the control group.

Studies have shown that gender differences affect the correlation between candidate genes and schizophrenia (Hoenicka et al., 2010). We evaluated the association of the detected SNPs with schizophrenia risk using gender as a classification criterion conducting by chi‐square test, as shown in Tables 3 and 4. We found that in the male population, the C allele (*p* = 0.015) and C/C genotype frequency (*p* = 0.037) of rs7116768 may be associated with schizophrenia susceptibility. However, when a Bonferroni correction was applied to mitigate against the so‐called “multiple comparison problem” (where for a significant *p*‐value of 0.5, 5% of tests are likely to be significant by chance), no significances were found. We do not have enough statistical power to detect any significant difference in the overall sample size and separately for men and women by the standards of 0.8.

**Table 3:** Genotype and allele frequencies of *DRD2* SNPs in control male subjects and schizophrenia male patients

SNP	Case		Control		*p*‐Value	OR	95% CI	Power
	*N*	%	*N*	%				
Rs7116768					0.037			
G/G	131	85.06	132	84.08				
G/C	14	9.10	23	14.65				
C/C	9	5.84	2	1.27				
G/G + G/C	145	94.16	155	98.73	0.034	0.208	0.044–0.978	0.586
C allele	32	10.39	54	8.60	0.015	1.791	1.121–2.863	0.625
Rs1047479195					0.576			
C/C	143	92.86	150	95.54				
C/A	9	5.84	5	3.18				
A/A	2	1.30	2	1.27				
A/A + C/A	11	7.14	7	4.46	0.341	1.648	0.622–4.370	0.180
A allele	13	4.22	9	2.87	0.392	0.670	0.282–1.590	0.117
Rs1799732					0.075			
Ins/Ins	131	85.06	132	84.08				
Ins/Del	15	9.74	23	14.65				
Del/Del	8	5.19	2	1.27				
Ins/Ins + Ins/Del	146	94.81	155	98.73	0.059	0.235	0.049–1.127	0.500
Del allele	64	10.06	54	8.60	0.262	0.792	0.530–1.184	0.121
Rs1799978					0.120			
A/A	109	70.78	104	66.24				
G/A	36	23.38	39	24.84				
G/G	9	5.84	14	8.92				
A/A + G/A	145	94.16	143	92.86	0.387	1.577	0.662–3.760	0.153
G allele	54	17.53	67	21.34	0.265	1.276	0.856–1.901	0.251

Note

The SNPs with minor allele frequency <0.01 were excluded. The *p*‐value was calculated by 2 × 3 and 2 × 2 chi‐square test, in which the codominant model, the recessive model, and the allele model were corrected by Bonferroni's correction and the *p < 0.05/4*was statistically significant. The statistical power is considered to be enough when power >0.8. The false discovery rate <0.05.

**Table 4:** Genotype and allele frequencies of *DRD2* SNPs in control female subjects and schizophrenia female patients

SNP	Case		Control		*p*‐Value	OR	95% CI	Power
	*N*	%	*N*	%				
Rs7116768					0.373			
G/G	126	82.89	143	85.63				
G/C	19	12.50	21	12.57				
C/C	7	4.61	3	1.80				
G/G + G/C	145	95.39	164	98.20	0.202	0.379	0.096–1.492	0.305
C allele	33	10.86	27	8.08	0.277	0.722	0.423–1.232	0.178
Rs1047479195					0.485			
C/C	141	92.86	156	93.41				
C/A	11	5.84	9	5.39				
A/A	0	1.30	2	1.20				
A/A + C/A	11	7.24	11	6.59	0.829	0.904	0.380–2.149	0.055
A allele	11	3.62	13	3.89	1.000	1.079	0.476–2.445	0.054
Rs1799732					0.373			
Ins/Ins	126	82.89	143	85.63				
Ins/Del	19	12.5	21	12.57				
Del/Del	7	4.61	3	1.80				
Ins/Ins +Ins/Del	145	95.39	164	98.20	0.202	0.379	0.096–1.492	0.374
Del allele	33	10.86	27	8.08	0.277	0.722	0.423–1.232	0.178
Rs1799978					0.710			
A/A	100	65.79	105	62.87				
G/A	43	28.29	48	28.74				
G/G	9	5.92	14	8.38				
A/A + G/A	143	94.08	153	91.62	0.517	1.454	0.610–3.463	0.133
G allele	61	20.07	76	22.75	0.441	1.173	0.803–1.716	0.139

Note

The SNPs with minor allele frequency <0.01 were excluded. The *p*‐value was calculated by 2 × 3 and 2 × 2 chi‐square test, in which the codominant model, the recessive model, and the allele model were corrected by Bonferroni's correction and the *p < 0.05/4*was statistically significant. The statistical power is considered to be enough to detect any significant difference when power >0.8. The false discovery rate <0.05.

### 3.2 Linkage disequilibrium and haplotypes

**Figure 1:**
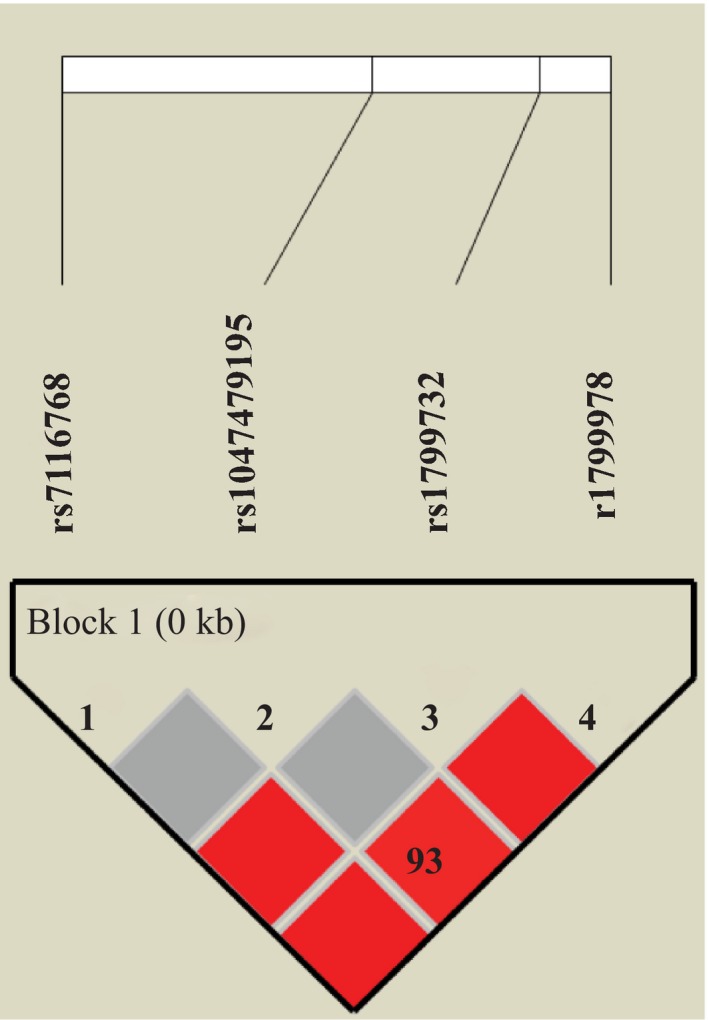
Linkage disequilibrium block composed by rs7116768, rs1799732, rs1047479195, and rs1799978. The number is the value of multiallelic *D*′, which represents the level of recombination between the two blocks

We employed Haploview 4.2 program to assess the Linkage disequilibrium (LD) block and haplotypes of the four SNPs. Among rs7116768, rs1799732, rs1047479195, and rs1799978, an LD block was made in Figure 1 (rs7199732 and rs7116768 *D*′ = 1.0, *r* ^2^ = 1.0; rs1799978 and rs7116768 *D*′ = 1.0, *r* ^2^ = 0.026; rs7199978 and rs7116768 *D*′ = 0.934, *r* ^2^ = 0.108; rs1799978 and rs1047479195 *D*′ = 1.0, *r* ^2^ = 0.026).

The relationship of haplotype distribution with schizophrenia was also analyzed. We found no association for both haplotype and the risk of schizophrenia (shown in Table 5).

**Table 5:** Haplotype analysis of *DRD2* SNPs in control subjects and schizophrenia patients

Haplotype	SNP				Case		Control		*p*‐Value	OR	CI
	Rs7116768	Rs1047479195	Rs1799732	Rs1799978	*N*	%	*N*	%			
1	G	C	insC	A	216	70.6	225	69.4	0.794	1.056	0.751–1.485
2	G	C	insC	G	46	14.9	61	18.8	0.243	0.763	0.502–1.160
3	C	C	delC	A	32	10.5	27	8.3	0.412	1.285	0.750–2.200

Note

Haplotype with frequency <0.05 was excluded.

## 4 DISCUSSION

In the present study, we found both of the rs7116768 and rs7199732 have a statistical difference between schizophrenic patients and control group. Nevertheless, after the Bonferroni correction, both of the observed associations with schizophrenia tended to disappear. Similarly, we did not find any association between *DRD2* promoter polymorphisms and schizophrenia in the gender groups, which are consistent with the study of Xiao et al. (2013). SNP is a bimodal genetic marker for low heterozygosity. In order to improve the heterozygosity and to use the genetic information more effectively, we performed haplotype analysis. And no statistical difference was found in all of the four haplotypes between schizophrenia patients and control subjects.

Previous studies mainly focused on the relationship between *DRD2* gene and schizophrenia were largely limited to coding regions (Liu et al., 2014; Liu, Liu, An, Zhang, & Wang, 2012; Yang et al., 2016) and showed that the *DRD2* gene coding region polymorphisms of rs1076560, rs6277, and so on were associated with schizophrenia in the Chinese Han population (Fan et al., 2010; Zheng, Shen, & Xu, 2012). As for the promoter region of *DRD2* gene, previous studies were mainly focused on rs1799732 (shown in Table 6). Our results are also lining with previous studies which mostly were negative studies (Parsons et al., 2007; Stober et al., 1998; Tallerico, Ulpian, & Liu, 1999), except for several positive ones. As for the studies with positive findings, China (Peng, Wang, Cheng, Zhang, & Jiang, 2005) and Brazil (Cordeiro, Siqueira‐Roberto, Zung, & Vallada, 2009) showed that *del C* allele was a protective factor while Scotland (Breen et al., 1999) had an adverse conclusion.

**Table 6:** Previous studies on the association with rs1799732 and the etiology of schizophrenia

Author (Year)	Region	SNP	Sample size (case/control)	*p*‐Value
Breen et al. (1999)	Scotland	rs1799732	439/437	0.02
Peng et al. (2005)	China	rs1799732	120/100	<0.05
Cordeiro et al. (2009)	Brazil	rs1799732	229/733	0.001
Xiao et al. (2013)	China	rs1799732	120/100	<0.05
Dubertret et al. (2010)	France	rs1799732	108 trios	0.991
Rohrmeier et al. (2003)	Germany	rs1799732	190 trios	0.696
Himei et al. (2002)	Japan	rs1799732	190/103	0.520
Behravan et al. (2008)	Iran	rs1799732	38/63	0.94
Parsons et al. (2007)	Spain	rs1799732	119/165	>0.05

These inconsistent results may be due to the use of case–control study in these studies. The method is used widely because of its convenience and precision positioning. However, the approach is vulnerable to the effect of racial stratification, sample population, and sample content. Sample population issues may also explain the inconsistencies between our results and some meta‐analyses conducted in Asia (He et al., 2016; Wang et al., 2016) and even in China (Zhao et al., 2016). China is a vast country with a great geographical distance between north and south. In previous studies, most of the samples were from southern China, such as Wuhan (Peng et al., 2005; Xiao et al., 2013). We cannot exclude the influence of population structure differences on the results, so these conclusions cannot fully represent the situation of Han population in north China. This study can provide references for the association between *DRD2* promoter region and schizophrenia in the Han population in north China. The case–control study based on blood relationship is more referable because it excludes the influence factors of the population admixture (Kazeem & Farrall, 2005) such as French (Dubertret et al., 2010) and Germanic (Rohrmeier et al., 2003) family studies, they also end up at negative findings. Himei et al. (2002) found no association for several *DRD2* gene polymorphisms with schizophrenia and a severe association of positive symptoms of patients with the −141 C Del allele by studying the association with each polymorphism and PANSS score. All of these evidences implied that rs1799732 may not be directly related to the occurrence of schizophrenia, but through its del C allele affects the symptoms of schizophrenia patients.

Related to rs7116768, there was no reported study involved the association with this SNP locus and schizophrenia. Our study found that rs7116768 was slightly associated with schizophrenia in the northern Chinese Han population. Rs7116768 locates in 5'UTR in *DRD2* gene, and the base sequence of this region is transcribed for RNA without being translated into amino acids. The transcriptional products are removed during the modification of mature mRNA. This SNP site may be in a target region of transcription factors and cis‐regulatory elements. It may influence the expression level of *DRD2* gene by regulating the expression and stability of mRNA, and then change the activity of dopamine neurotransmitters to lead the onset of schizophrenia. The number of samples of this study is not sufficient for significantly reflecting the association between this polymorphic site and the risk of schizophrenia. Usage of Bonferroni correction in this study may increase the false‐negative rate. However, to fully explore the association with rs7116768 and schizophrenia, much more data are needed.

## 5 CONCLUDING REMARKS

In this study, we found no association for *DRD2* gene promoter region with schizophrenia risk in the northern Chinese Han population. But we cannot definitively exclude the possible association between *DRD2* gene promoter region and schizophrenia risk. Additional studies of rs7116768 and other *DRD2* SNPs will be required on a large data set. We hope our study data can provide a reference for future research.

## CONFLICT OF INTEREST

The authors report no conflicts of interest in this work.

## AUTHOR CONTRIBUTIONS

X.Z. wrote the initial manuscript. X.Z., A.A., Y.L., Y.L., and J.Y. conducted the experiment. X.Z., J.Y., J.X., J.X., M.D., A.A., and B.W. analyzed the results. A.A modified the manuscript. All authors reviewed the manuscript.

## ETHICAL APPROVAL

All participants gave their informed consent in writing after the study aims and procedures were carefully explained to them in their own language. The study was approved by the ethical review board of the China Medical University, Shenyang Liaoning Province, People's Republic of China and in accordance with the standards of the Declaration of Helsinki.
